# Supplementary material for: Novel Computed Tomography Variables for Assessing Tricuspid Valve Morphology: Results from the TRIMA (Tricuspid Regurgitation IMAging) Study
Source: J Clin Med. 2022 May 17;11(10):2825. doi: 10.3390/jcm11102825 (PMC9143522; doi:10.3390/jcm11102825)
Supplement: Supplementary file 1 [file jcm-11-02825-s001.zip › jcm-1647908-supplementary.pdf]

Journal of
Clinical Medicine

Supplementary Material

Automated TV annulus area and perimeter<br>Manual commissure identification<br>(Tricuspid valve workflow)	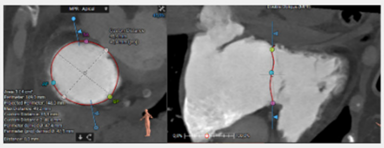
Septal-lateral and the antero-posterior annular dimensions<br>(Tricuspid valve workflow)	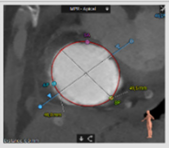
Distance between commissures<br>(Tricuspid valve workflow)	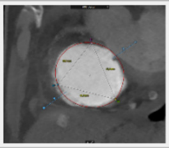
Identification of the geometrical centroid<br>(Tricuspid valve workflow)	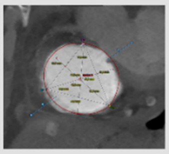
Distance between the centroid and commissures<br>(Tricuspid valve workflow)	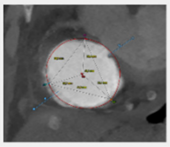
Angles formed by the centroid and two contiguous commissures<br>(Tricuspid valve workflow)	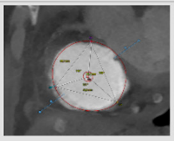
Tenting height in 4ch and 2ch<br>(MPR)	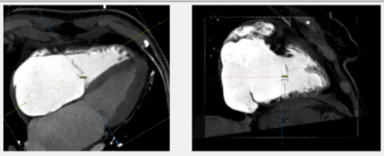
Leaflets tethering degree<br>(MPR)	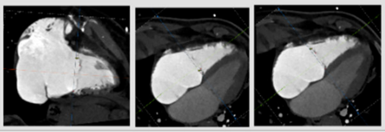
AROA<br>(MPR)	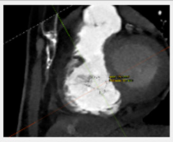
RA and RV height in 4ch<br>(MPR)	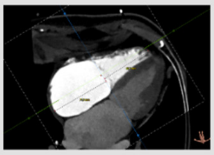
RA and RV height in 2ch<br>(MPR)	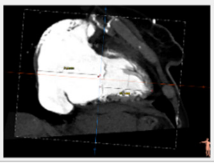

Figure S1. Step-by-step analysis using the 3mensio Structural Heart software (Pie Medical Imaging, Maastricht, The Netherlands). MPR: multiplanar reconstruction; AROA: anatomical regurgitant orifice area; RA: right atrium; RV: right ventricle; 4ch: four-chamber; 2ch: two-chamber.
